# Microbial Composition in the Duodenum and Ileum of Yellow Broilers With High and Low Feed Efficiency

**DOI:** 10.3389/fmicb.2021.689653

**Published:** 2021-07-27

**Authors:** Huijiao Lv, Yun Huang, Tao Wang, Shangkun Zhai, Zhuocheng Hou, Sirui Chen

**Affiliations:** National Engineering Laboratory for Animal Breeding and MOA Key Laboratory of Animal Genetics and Breeding, College of Animal Science and Technology, China Agricultural University, Beijing, China

**Keywords:** yellow broilers, intestinal microbiota, feed efficiency, ileum, duodenum, 16S rRNA

## Abstract

The composition of the gut microbiome plays important roles in digestion, nutrient absorption, and health. Here, we analyzed the microbial composition in the duodenum and ileum of yellow broilers. Chickens were grouped based on feed efficiency (high feed efficiency [HFE] and low feed efficiency [LFE] groups; *n* = 22 each). Microbial samples from the duodenum and ileum were collected, and *16S* rRNA sequencing of the V3–V4 region was performed. The dominant bacteria in the duodenum were from the phyla Firmicutes and Cyanobacteria and the genera *Lactobacillus*, *Faecalibacterium*, and *Ruminococcus*. In the ileum, the phyla Firmicutes and Proteobacteria and the genera *Lactobacillus*, *SMB53* and *Enterococcus* were predominant. Alpha diversity analysis showed that the microbiota diversity was significantly higher in the duodenum than in the ileum. The structure of the ileal microbiota was similar between groups, and the species richness of the microbiota in the HFE group was significantly higher than that in the LFE group. In the HFE and LFE groups, Firmicutes and Cyanobacteria were negatively correlated, and *Lactobacillus* had medium to high negative correlations with most other genera. Functional prediction analysis showed that the gluconeogenesis I pathway was the most abundant differential metabolic pathway and was significantly altered in the LFE group. Moreover, although the microbial community structures were similar in the duodenum and ileum, the diversity of the microbial community was significantly higher in the duodenum than in the ileum. Pearson correlation analysis revealed that the phylum Chloroflexi and genera *Acinetobacter*, *Pseudomonas*, *Bacillus* and *Neisseria* were with coefficients <−0.3 or >0.3. In the ileum, *Ruminococcus* may be associated with HFE whereas *Faecalibacterium* may be associated with LFE. These findings may provide valuable foundations for future research on composition and diversity of intestinal microbes and provide insights into the roles of intestinal microbes in improving feed efficiency and the industrial economic benefits of yellow broilers.

## Introduction

Feedstuff costs occupy approximately 70–80% of the total cost of chicken feeding; thus, feed efficiency (FE) and body weight (BW) gain (BWG) are commonly used to measure the growth performance of poultry (Aggrey et al., 2010; Singh et al., 2012). The production performance of broilers is dependent on many factors, including heredity, diet, age, and microorganisms (Havenstein et al., 2003; Lu et al., 2003; Aggrey et al., 2010). Recent studies on the chicken gut microbiota have shown that intestinal microbes can provide a large number of enzymes and substrates, thereby affecting the FE, absorption, and immune function of the host (Stanley et al., 2013; Schokker et al., 2015).

Understanding the role of the gut microbiome in determining FE and BWG is important for human health and animal science. In humans, the focus has been on how gut microbes can aid on reducing obesity, whereas in animal production, the aim is to identify microbes that can effectively convert food into weight gain, particularly muscle gain (Stanley et al., 2016). The stability of the intestinal microbiota not only contributes to poultry health but also plays important roles in improving the economic benefits of the poultry industry.

The microbiota in the gastrointestinal tract is complex and has an effect on maintaining intestinal health, influencing digestion, and affecting the overall production performance of chickens (Shang et al., 2018). Feed conversion ratio (FCR) is widely used to estimate feed efficiency (yield per unit feed), described as the ratio between feed inputs and product outputs during the measurement period (Siegel, 2014; Sell-Kubiak et al., 2017; Lima et al., 2019). Singh et al. (2012) reported that *Acinetobacter*, *Arcobacter*, and other microorganisms are predominant in broilers with a high FCR, whereas microbes such as *Barnesiella* and *Cloacibacillus* are abundant in broilers with a low FCR. The genus *Lactobacillus* has been demonstrated to be related to FCR in chickens (Torok et al., 2011; Stanley et al., 2016). Torok et al. (2011) reported *Lactobacillus salivarius*, *L. aviarius*, and *L. crispatus* in the ileum and showed that the presence of these microbes contributed to a low FCR in broilers. Moreover, Stanley et al. (2016) also found that the presence of *Lactobacillus* sp. resulted in a low FCR, whereas the presence of the genus *Faecalibacterium* increased the FCR and gain rate. In addition to intestinal microorganisms, the fecal microbiome has been shown to be related to feed conversion in broiler breeders (Díaz-Sánchez et al., 2019).

Research on the structure and variation of the intestinal microbiota in poultry can provide a theoretical basis for improving FE and growth performance and promoting the development of microbial biomass resources and preparations, which are essential for improving industrial efficiency. A diet with *Lactobacillus* strains was found to increase BWG and FE and decrease mortality in broilers (Zulkifli et al., 2000; Timmerman et al., 2006). Fonseca et al. (2010) reported that supplementation with probiotics could reduce the quantity of enterobacteria and pathogenic bacteria in the cecum of broiler chickens.

Small intestine, which includes duodenum, jejunum and ileum, is the major place for nutrition digestion and absorption. In addition, fermentation process is mainly in the cecum. Microbial density and diversity were found to be greatest in the cecum (Rehman et al., 2007). Fecal samples were easy to obtain and usually used as substitutes for gut microbes (Wen et al., 2021). Studies on the relationship between FE and gastrointestinal microbes in broilers have mainly focused on microbes in the cecum and feces, whereas few studies have focused on those in the duodenum and ileum. Therefore, the aim of this study was to explore the microbial composition of and differences in the duodenum and ileum of yellow broilers and determine the relationships of microorganisms in the duodenum and ileum with FE via *16S* rRNA sequencing. Our findings may provide valuable foundations for future research on intestinal microbiota and FE in broilers.

## Materials and Methods

### Ethics Statement

All animal experiments followed the principles formulated by the Ministry of Agriculture, China. Ethics approval for this study was obtained from the Animal Care Committee of China Agricultural University.

### Animals and Samples

In total, 270 male yellow broilers were raised at the breeding farm of Jiangsu Xingmu Agricultural Science and Technology Co. (Beijing, China). Each chicken was raised in an independent cage with a food conditioner in the same environment from hatching to 63 days. The feeding experiment was divided into three stages: from day 1 to 20, from day 21 to 40, and from day 41 to 63. The feed formula (Supplementary Table 1) was the same as that described by Huang et al. (2021) and met the nutritional requirements set forth in the Nutritional Requirements of Chickens (1994). The feed intake and BW per chicken were measured every 5 days. The FCR was calculated as the ratio of FE to BWG during days 5 to 63 of feeding. According to the FCR ranking, 22 chickens with high FCR were assigned to the high FE (HFE) group and 22 chickens with low FCR were assigned to the low FE (LFE) group.

On day 64, chickens from each group were euthanized, and samples of the duodenum and ileum were aseptically collected. The samples were stored at −80°C. Microbial genomic DNA was extracted and purified using a Mag-Bind Stool DNA Kit (Omega Bio-Tek, Norcross, GA, United States) according to the manufacturer’s instructions. The quality and quantity of the purified DNA were verified using a NanoDrop spectrometer (Thermo Fisher Scientific, Waltham, MA, United States).

### 16S rRNA Gene Amplicon Sequencing

The V3–V4 hypervariable region of the barcoded *16S* rRNA was sequenced at Beijing igeneCode Biotech Co., Ltd. (Beijing, China). Polymerase chain reaction (PCR) was performed using Phusion Master Mix with the forward primer 341F (5′-ACTCCTACGGGAGGCAGCAG-3′) and reverse primer 806R (5′-GGACTACHVGGGTWTCTAAT-3′). The reaction system consisted of 25 μL 2 × Phusion Master Mix, 2.5 μL forward primer, 2.5 μL reverse primer, 30 ng template DNA, and water to bring it to reaction volume of 50 μL. The reaction conditions were as follows: 94°C for 3 min; 30 cycles of denaturation at 95°C for 30 s, annealing at 56°C for 45 s, extension at 72°C for 45 s, and extension at 72°C for 10 min. The library was constructed using amplicons and qualified using an Agilent 2100 Bioanalyzer (Agilent Technologies, Santa Clara, CA, United States). Paired-end sequencing was performed using an Illumina HiSeq2500 platform (Illumina, San Diego, CA, United States) and generated 250 bp paired-end reads. The datasets presented in this study can be found in the National Center for Biotechnology Information (NCBI) database with the accession number PRJNA721286.

### Quality Control and Processing

Raw data were filtered (Ewing et al., 1998; Li and Durbin, 2009; Fadrosh et al., 2014) as follows: (1) removal of sequence reads with average quality less than 20 under the sliding window of 30 bp; (2) removal of reads with missing length greater than 25% of the original reads; (3) removal of the reads with more than 3 bp mismatch with adapter; (4) removal of the reads with “N.” Clean data were obtained after filtering raw data and were split according to barcoded sequences. Clean data for each sample were then assembled with fast length adjustment of short reads (FLASH, V1.2.11) (Magoč and Salzberg, 2011). The minimum matching length was 15 bp and the allowable mismatch ratio of overlapping area was 0.1. Reads without overlap relationship were all removed. Clean tags were clustered using UPARSE software (Edgar, 2013) with 97% similarity standard to generate a representative sequence of operational taxonomic units (OTUs). The chimeras generated by PCR amplification were compared with the Gold database (v20110519) using UCHIME (v4.2.40) software (Edgar et al., 2011) and removed from the representative sequences of OTUs with default parameters. The abundance of each OTU in each sample was obtained using USEARCH (v7.0.1090) software (Quast et al., 2013). The OTU representative sequences were annotated according to the GreenGene database (DeSantis et al., 2006) using the RDP classifier (v2.2) with QIIME (v1.9.1) software and a confidence threshold was of 0.8. After obtaining microbial classification information, the relative abundance of microorganisms in each sample was analyzed at different classification levels.

### Composition Analysis of the Intestinal Microbiota and Statistical Analysis

Linear discriminant analysis effect size (LEfSe) analysis was performed to identify microbial species with significant differences between groups. Based on non-parametric Kruskal–Wallis and rank tests, linear discriminant analysis (LDA) was used to evaluate the influence of species with significant differences, and communities having significant effects (LDA > 2) on sample division were determined (Segata et al., 2011). Alpha diversity (including observed species, Chao1, Ace, Shannon Wiener and Simpson indexes) and beta diversity (principal co-ordinates analysis, PCoA) were analyzed using Mothur (v1.31.2) and QIIME software, respectively. Analysis of similarities (ANOSIM) was performed using the “vegan” R package to assess whether the intergroup difference was significantly greater than that within the group. Microbial community function was predicted for all sample data using PICRUSt2 (Douglas et al., 2019). Statistical analysis of phenotypic data and *t*-tests of FCR, average daily feed intake, and average BW of samples were processed using Microsoft Excel. Correlations between FCR and taxonomic relative abundance at the phylum and genus levels were analyzed using Pearson correlation coefficients, and significance tests were performed using the “ggcorrplot” package in R; results with *P*-values ≤ 0.05 were considered statistically significant. The relative abundances of OTUs were statistically analyzed using Wilcoxon rank sum tests in R, and the obtained *P*-values were corrected using the “p.adjust” function of the “BH” method in R. The statistical method of Welch’s *t*-test in STAMP software was used to analyze differences between the HFE and LFE groups for the predicted results in the MetaCyc functional pathway.

The relative abundances of part of the microorganisms in the HFE and LFE groups were statistically analyzed using the non-parametric Wilcoxon rank-sum test, and false discovery rate (FDR) correction performed to explore differences in microbial composition at the phylum and genus levels for the two FE groups.

## Results

### Phenotypic Data and Sequencing

Body weight gain, feed intake, and FCR for the HFE and LFE groups are presented in Table 1 and Supplementary Figure 1. The results showed that the FCR values for the HFE and LFE groups were significantly different, allowing us to apply these conditions for subsequent experiments. The detailed ingredients and nutrient composition of diets were shown in Supplementary Table 1. The number of sample sequences for the duodenum ranged from 55,824 to 64,026, and the average numbers of sample sequences was 62,986 for the HFE group and 63,493 for the LFE group. The number of sample sequences for the ileum ranged from 53,568 to 63,743, and the average numbers of sample sequences was 62,612 for the HFE group and 63,214 for the LFE group. The OTU distribution of each group and OUT rank curve for each sample are shown in Supplementary Figure 2. The average number of OTUs in the duodenum and ileum samples was 297 and 175, respectively.

**TABLE 1:** Descriptive statistics of samples.

	**HFE group**	**LFE group**	***P*-value**
*N*	22	22	/
Average daily feed intake (g)	103.29 ± 5.66	119.52 ± 6.29	<0.01
BWG (g)	2883.42 ± 154.84	2846.73 ± 134.98	0.42
FCR	2.08 ± 0.05	2.44 ± 0.05	<0.01

*N, number of samples; BWG, body weight gain from day 5 to day 63; FCR, feed conversion ratio; HFE, high feed efficiency; LFE, low feed efficiency.*

### Composition Analysis of Microbiota in the Duodenum and Ileum

**FIGURE 1:**
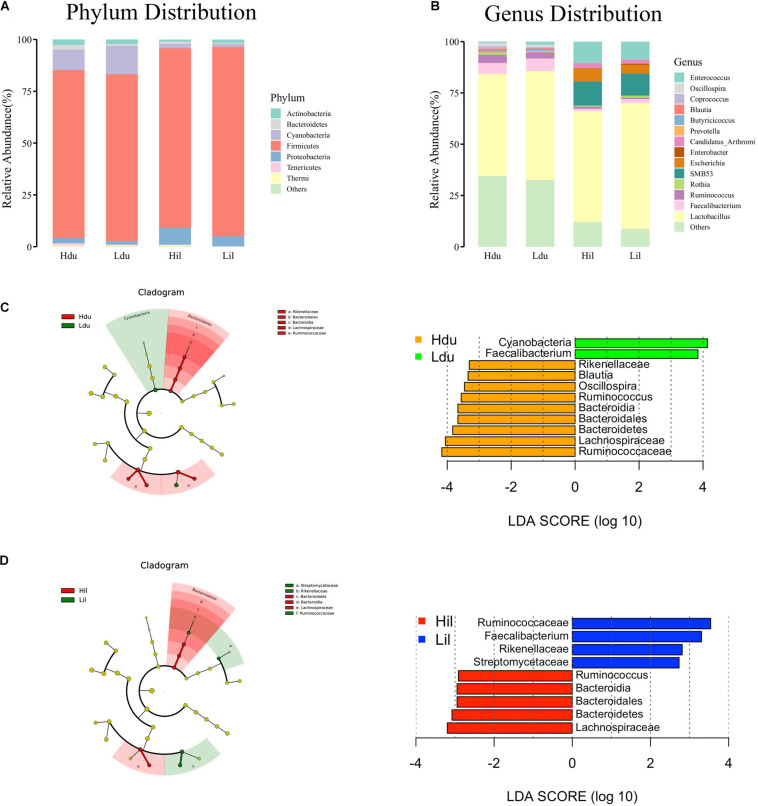
Analysis of microbial community structure of the duodenum and ileum at phylum level **(A)** and genus level **(B)**. Each bar represents the average relative abundance of each bacterial taxon in corresponding taxon. Cladogram and LDA value distribution histogram of duodenum **(C)** and ileum **(D)**. Hdu and Ldu, HFE group and LFE group of the duodenum. Hil and Lil, HFE group and LFE group of the ileum.

A total of 17 phyla and 50 genera were detected in the duodenum and ileum respectively, and were used for subsequent analysis. Microbial correlations at phylum and genus level in the HFE and LFE groups are shown in Figures 1A,B, respectively. Moreover, the specific values are shown in Supplementary Table 2. The phyla Firmicutes, Cyanobacteria, Actinobacteria, Proteobacteria, and Bacteroidetes, had higher relative abundance and were the dominant bacteria in both groups in the duodenum and ileum, accounting for over 99% of the microbial community. The proportion of each phylum in each sample fluctuated greatly, particularly for Firmicutes and Cyanobacteria; however, these two phyla constituted approximately 90% of the total bacterial community. Statistical analysis of the main phyla and genera of the duodenum showed no significant differences between groups, indicating that the microbial structure compositions at the phylum and genus levels were similar for the HFE and LFE groups in the duodenum. The relative abundances of phyla Bacteroidetes, Thermi and Tenericutes and genera *Faecalibacterium*, *Ruminococcus*, *Thermus*, *Oscillospira*, *Coprococcus* and *Butyricicoccus* in the ileum showed significant differences. The genera *Lactobacillus*, *Faecalibacterium*, *Ruminococcus*, *Oscillospira*, *Coprococcus*, and *Enterococcus* were the dominant bacterial genera in the duodenum, and the ratio of each genus varied between the two groups. The genera *Lactobacillus*, *SMB53*, *Enterococcus*, *Candidatus_Arthromitus*, *Escherichia*, and *Faecalibacterium* were dominant in the ileum, although the proportion of each genus differed between the HFE and LFE groups. The genera *Lactobacillus*, *Faecalibacterium*, and *Enterococcus* were dominant in both the duodenum and ileum of the HFE and LFE groups.

LEfSe method was used to distinguish bacterial taxa between HFE and LFE groups in the duodenum) and ileum, with the respective cladograms showing the differences in taxa of the structure of the duodenal (Figure 1C) and ileal (Figure 1D) microbiota and the predominant bacteria between HFE and LFE groups. Figure 1C shows that Cyanobacteria and *Faecalibacterium* were higher enriched in the LFE group, while Rikenellaceae, *Blautia*, *Oscillospira*, *Ruminococcus*, Bacteroidia, Bacteroidales, Bacteroidetes, Lachnospiraceae and Ruminococcaceae were more enriched in the HFE group in the duodenum. Figure 1D shows that the relative abundance of *Ruminococcus*, Bacteroidia, Bacteroidales, Bacteroidetes and Lachnospiraceae were higher in the HFE group, whereas the relative abundance of *Ruminococcaceae*, *Faecalibacterium*, Rikenellaceae and Streptomycetaceae were higher in the LFE group in the ileum. Pearson correlation tests were used to analyze the microbiota at the phylum and genus levels in the HFE and LFE groups. The phyla Firmicutes and Cyanobacteria in the duodenum of the HFE (*r* = −0.92) and LFE (*r* = −0.97) groups showed significant negative correlations (*P* < 0.01). In the duodenum, the genus *Lactobacillus* was significantly negatively correlated with most genera, and the correlation was moderate to high (Table 2). In the ileum, the relative abundances of the phyla Bacteroidetes, Thermi, and Tenericutes in the HFE group were significantly higher than those in the LFE group (*P* < 0.05). There was no significant difference in the relative abundance of ileal *Lactobacillus* between the HFE (54.09%) and LFE (61.28%) groups. The results of bacterial correlation analysis at the genus level are shown in Table 3.

**TABLE 2:** Correlation coefficient and significance of part of the microbes at genus level in the duodenum of HFE and LFE groups.

**Genus**		***Lactobacillus***	***Enterococcus***	***Bacteroides***	***Bifidobacterium***	***Blautia***	***Butyricicoccus***	***Coprococcus***	***Faecalibacterium***	***Oscillospira***	***Ruminococcus***	***SMB53***
*Lactobacillus*	H		0.368	−0.430	−0.144	−0.534	−0.520	−0.533	−0.556	−0.709	−0.594	−0.230
	L		−0.339	−0.455	−0.433	−0.580	−0.737	−0.774	−0.702	−0.714	−0.752	−0.392
*Enterococcus*	H	0.092		−0.157	−0.153	−0.367	−0.328	−0.233	−0.313	−0.302	−0.222	0.058
	L	0.123		−0.018	−0.012	0.398	0.272	0.210	0.333	0.147	0.050	−0.145
*Bacteroides*	H	0.046	0.486		−0.013	0.515	0.451	0.915	0.029	0.554	0.033	0.076
	L	0.033	0.938		−0.148	0.298	0.693	0.449	0.385	0.661	0.456	0.832
*Bifidobacterium*	H	0.523	0.496	0.953		−0.001	0.432	0.066	0.376	0.202	0.358	0.375
	L	0.044	0.958	0.511		0.227	0.156	0.539	0.375	0.284	0.465	0.163
*Blautia*	H	0.011	0.093	0.014	0.996		0.805	0.629	0.591	0.868	0.502	0.182
	L	0.005	0.067	0.178	0.309		0.525	0.743	0.636	0.784	0.381	0.255
*Butyricicoccus*	H	0.013	0.137	0.035	0.045	6.14E-06		0.668	0.674	0.81	0.574	0.233
	L	9.19E-05	0.221	3.54E-04	0.487	0.012		0.785	0.875	0.855	0.833	0.634
*Coprococcus*	H	0.011	0.296	2.52E-09	0.771	0.002	6.86E-04		0.326	0.694	0.302	0.045
	L	2.37E-05	0.348	0.036	0.01	7.58E-05	1.52E-05		0.829	0.909	0.799	0.497
*Faecalibacterium*	H	0.007	0.156	0.897	0.085	0.004	5.76E-04	0.139		0.715	0.923	0.154
	L	2.68E-04	0.13	0.077	0.086	0.001	1.03E-07	1.83E-06		0.812	0.808	0.411
*Oscillospira*	H	2.22E-04	0.173	0.007	0.366	1.61E-07	4.86E-06	3.42E-04	1.83E-04		0.717	0.215
	L	1.89E-04	0.513	8.18E-04	0.201	1.58E-05	4.07E-07	5.05E-09	4.52E-06		0.784	0.665
*Ruminococcus*	H	0.004	0.321	0.883	0.102	0.017	0.005	0.171	9.35E-10	1.72E-04		0.373
	L	5.50E-05	0.825	0.033	0.029	0.08	1.48E-06	8.24E-06	5.39E-06	1.57E-05		0.538
*SMB53*	H	0.303	0.799	0.735	0.086	0.417	0.297	0.842	0.494	0.336	0.087	
	L	0.071	0.519	1.58E-06	0.47	0.252	0.002	0.019	0.057	7.32E-04	0.01	

*H, HFE group; L, LFE group; upper triangle, Pearson’s correlation coefficient; lower triangle, Corresponding significance *P*-value.*

**TABLE 3:** Correlation coefficient and significance of part of the microbes at genus level in the ileum of HFE and LFE groups.

		***Lactobacillus***	***Bacteroides***	***Bifidobacterium***	***Blautia***	***Butyricicoccus***	***Candidatus_Arthromitus***	***Coprococcus***	***Faecalibacterium***	***Oscillospira***	***Ruminococcus***
*Lactobacillus*	H		−0.357	−0.517	−0.339	−0.352	−0.197	−0.329	−0.441	−0.37	−0.327
	L		−0.19	−0.452	−0.53	−0.503	−0.212	−0.505	−0.486	−0.497	−0.506
*Bacteroides*	H	0.103		−0.001	0.426	0.318	0.177	0.492	0.499	0.664	0.408
	L	0.398		0.669	0.47	0.576	0.688	0.648	0.223	0.516	0.397
*Bifidobacterium*	H	0.014	0.996		−0.044	0.049	−0.176	−0.106	0.194	−0.045	−0.081
	L	0.035	6.56E-04		0.397	0.55	0.557	0.606	0.33	0.513	0.452
*Blautia*	H	0.123	0.048	0.846		0.955	0.623	0.99	0.935	0.932	0.974
	L	0.011	0.027	0.019		0.978	0.299	0.972	0.944	0.978	0.988
*Butyricicoccus*	H	0.108	0.149	0.827	5.60E-12		0.63	0.939	0.94	0.867	0.959
	L	0.017	0.005	0.008	4.28E-15		0.43	0.991	0.919	0.994	0.972
*Candidatus_Arthromitus*	H	0.38	0.431	0.433	0.002	0.002		0.636	0.589	0.473	0.662
	L	0.343	3.97E-04	0.007	0.177	0.046		0.44	0.117	0.351	0.231
*Coprococcus*	H	0.135	0.02	0.639	2.63E-18	9.85E-11	0.001		0.915	0.946	0.979
	L	0.016	0.001	0.003	4.78E-14	4.38E-19	0.04		0.88	0.981	0.953
*Faecalibacterium*	H	0.04	0.018	0.387	1.89E-10	8.15E-11	0.004	2.61E-09		0.917	0.915
	L	0.022	0.319	0.133	4.63E-11	1.65E-09	0.603	6.97E-08		0.949	0.979
*Oscillospira*	H	0.09	7.59E-04	0.841	3.03E-10	1.73E-07	0.026	2.86E-11	1.96E-09		0.895
	L	0.019	0.014	0.015	4.75E-15	1.54E-20	0.109	1.20E-15	1.70E-11		0.986
*Ruminococcus*	H	0.137	0.059	0.719	1.98E-14	2.09E-12	7.90E-04	3.10E-15	2.40E-09	1.89E-08	
	L	0.016	0.067	0.035	9.99E-18	4.00E-14	0.301	7.16E-12	2.59E-15	6.87E-17	

*H, HFE group; L, LFE group; upper triangle, Pearson correlation coefficient; lower triangle, Corresponding significance *P*-value.*

### Diversity Analysis of the Microbiota in the Duodenum and Ileum

**FIGURE 2:**
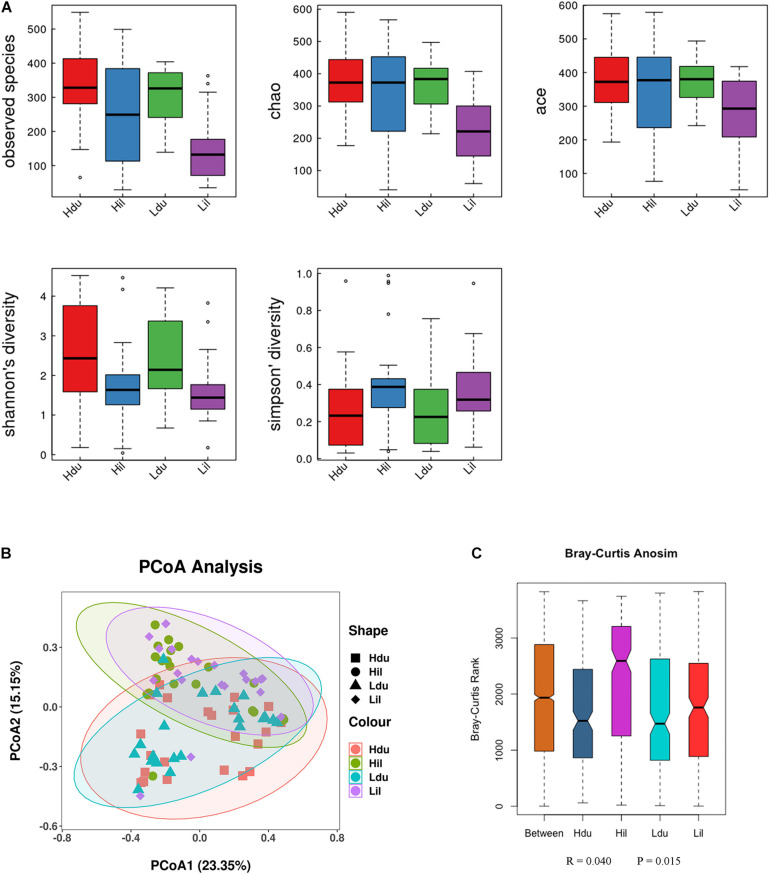
Differences in bacterial diversity, richness, and structures in the duodenum and ileum of HFE and LFE groups. **(A)** Differences in community diversity and richness between the duodenum and ileum. **(B)** Principal co-ordinates analysis (PCoA) plot of bacterial community structure between the duodenum and ileum. Hdu and Ldu, HFE group and LFE group of the duodenum. Hil and Lil, HFE group and LFE group of the ileum. **(C)** Plot of analysis of similarities (ANOSIM).

Alpha diversity (observed species, Chao1, Ace, Shannon Wiener and Simpson indexes) was measured to describe species richness and evenness (Walters and Martiny, 2020). The statistical results of alpha-diversity analysis of the duodenum and ileum in the HFE and LFE groups are shown in Figure 2A and Supplementary Table 3. The observed species, Chao1 index, and ACE index in the HFE group were significantly higher than those in the LFE group (*P* < 0.05), indicating that the microbial community richness of the ileum in the HFE group was higher than that in the LFE group.

Beta-diversity was used to compare differences in species diversity between multiple samples (Anderson et al., 2006). PCoA of the community structure between the HFE and LFE groups in the duodenum and ileum is shown in Figure 2B,C. (ANOSIM, *R* = 0.040, *P* = 0.015) showed that the differences between groups were greater than that within groups, which means that the grouping was effective. However, ANOSIM results demonstrated that the microbial communities were similar between HFE and LFE groups for the duodenum (ANOSIM, *R* = −0.031, *P* = 0.956) and ileum (ANOSIM, *R* = −0.010, *P* = 0.578).

### Correlation Analysis of FE With Duodenal and Ileal Microbiota

**FIGURE 3:**
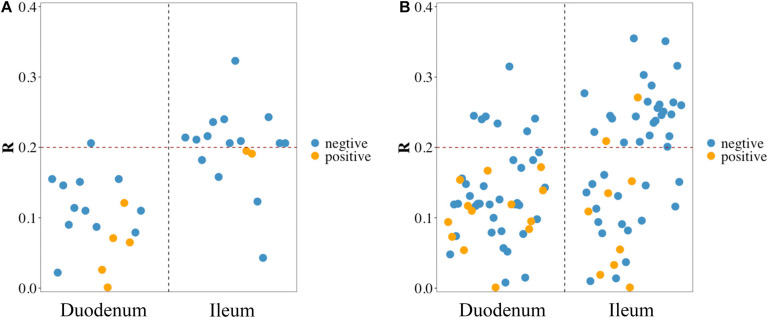
Pearson correlation between feed conversion ratio (FCR) and microbes in the duodenum and ileum at phylum level **(A)** and genus level **(B)**. Blue/yellow dot in scatter diagram: microbes in the corresponding intestinal segment negative/positive correlated with FCR.

Pearson correlation analysis was performed between FCR and 17 phyla (Figure 3A) and between FCR and 50 genera (Figure 3B) detected in the duodenum and ileum. The results showed that FE was negatively correlated with most microorganisms at the phylum level. The absolute values of correlation coefficients of phylum Chloroflexi and genera *Acinetobacter*, *Pseudomonas*, *Bacillus* and *Neisseria* were greater than 0.3 (Table 4). The number of microorganisms negatively correlated with FCR was greater than the number of microorganisms positively correlated with FCR in both the duodenum and ileum. Moreover, the correlation between FCR and genera Thermus (duodenum: 0.073; ileum: −0.241), Paenibacillus (duodenum: −0.244; ileum: 0.271) and Alcaligenes (duodenum: 0.119; ileum: −0.207) was opposite in the duodenum and ileum.

**TABLE 4:** Correlation coefficients of feed conversion ratio (FCR) and part of the microorganisms at phylum and genus levels.

**Taxa**	**Microbe**	**Duodenum**	**Ileum**
Phylum	Chloroflexi	−0.087	−0.323
Genus	*Acinetobacter*	−0.315	−0.355
	*Pseudomonas*	−0.234	−0.303
	*Bacillus*	−0.126	−0.351
	*Neisseria*	−0.119	−0.316

### Functional Prediction of Duodenal and Ileal Microbiota of the HFE and LFE Groups

**FIGURE 4:**
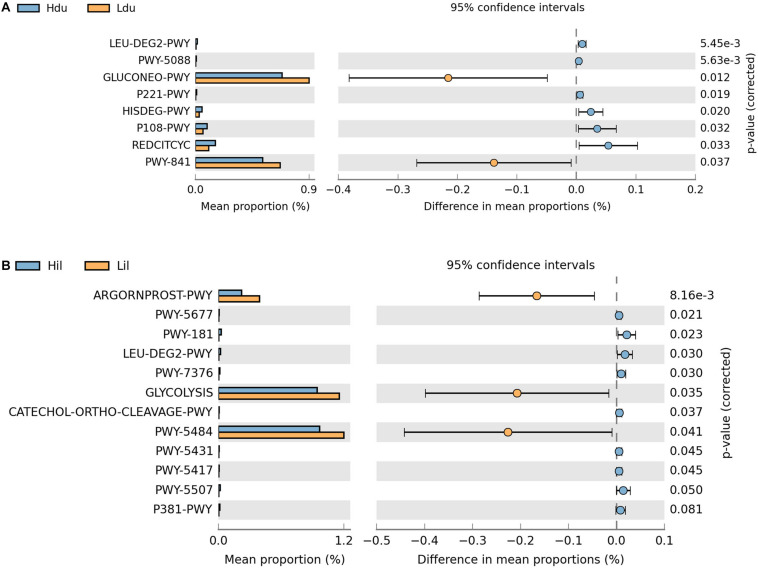
Microbial function prediction in the duodenum **(A)** and ileum **(B)** of HFE and LFE groups. The *P-*values were displayed on the far right. Hdu and Ldu, duodenum of HFE and LFE groups, respectively; Hil and Lil, ileum of HFE and LFE groups, respectively.

Next, we aimed to elucidate the potential functions of the microorganisms. In total, 401 and 403 MetaCyc metabolic pathways were predicted in the duodenum and ileum, respectively, using PICRUSt2. Pathways with an average relative abundance greater than 0.001% in the HFE and LFE groups are shown in Figure 4 and Supplementary Table 4. The proportion of microorganisms involved in glucose metabolic process in the duodenum of both groups was the highest among all differential pathways and was significantly enriched (*P* < 0.05) in the LFE group (Figure 4A). Moreover, the enrichment of superpathways of purine nucleotide *de novo* biosynthesis I, tricarboxylic acid cycle VIII (*Helicobacter*), octane oxidation, and pyruvate fermentation to propanoate I was significantly different between the HFE and LFE groups in the duodenum (*P* < 0.05). In the ileum (Figure 4B), pathways including glycolysis II (from fructose 6-phosphate); glycolysis I (from glucose 6-phosphate); and arginine, ornithine, and proline interconversion were significantly enriched in the LFE group (*P* < 0.05).

## Discussion

In this study, 44 yellow broilers divided into HFE and LFE groups were evaluated to determine the microbial composition and relationships of microorganisms in the duodenum and ileum with FE. Differences in duodenal and ileal microbial community structure and composition under HFE and LFE were studied using *16S* rRNA sequencing technology, and the differential metabolic pathways between the two groups were predicted.

The intestinal microbiota has been extensively studied in recent years and has been shown to interact broadly with the host through matrix metabolic exchange and co-metabolism (Nicholson et al., 2005). Some metabolic pathways in microbial communities fill the evolutionary gaps in the metabolic characteristics of some human and animal bodies, e.g., decomposition of insoluble plant polysaccharides (Gibson and Roberfroid, 1999), biotransformation of conjugated bile acids (Hylemon and Harder, 1998), and synthesis of certain vitamins (Hill, 1997). In humans, there are approximately 10^12^ parenchymal cells (including blood and nerve cells) in the body, whereas the number of bacteria in the intestine is approximately 10^14^, with a total weight of more than 1 kg, and the total genome of intestinal bacteria may be 100-times larger than the total genome of the human body (Xu and Gordon, 2003; Qin et al., 2010). This large number of microbial communities in the intestine results in the formation of a complex internal environment network. Host genes also affect the gut microbiota distribution in mammals (Mignon-Grasteau et al., 2015). A balanced microbial community structure can promote various biological processes in animals, including improving the ability of the intestine to resist pathogens and boost immunity (Xu and Gordon, 2003), promoting intestinal villus development (Samanya and Yamauchi, 2002), and fermenting and degrading dietary fibers that are hard to digest (Flint and Bayer, 2008).

Compared with the digestive tract of mammals, the digestive tract of chickens is relatively short; the retention time of nutrients in the digestive tract is also short, although this does not affect the digestive efficiency (Burt, 2007). This phenomenon may be related to the complex, diverse, and efficient gastrointestinal microorganisms in chickens (Sergeant et al., 2014). Diversified bacterial communities and functions in chicken intestine depend on the age of the bird, location of the bacteria in the gastrointestinal tract, and composition of the feed (Pan and Yu, 2014; Mancabelli et al., 2016).

Small intestine (consisting of the duodenum, jejunum and ileum) and cecum are major places for nutrition digestion, absorption and fermentation. Previous studies showed that the cecum has the greatest microbial density and diversity. Longer digestion time of nutrients in the cecum allows enhanced microbial fermentation (Rehman et al., 2007). Feces are easy to sample for subsequent analysis and are usually used as substitutes for intestinal microbiota (Wen et al., 2021). However, the heterogeneity of digestive tract function leads to the regional differences within the intestinal microbial populations (Martinez-Guryn et al., 2019). Microorganisms in the environment may also affect fecal samples, thus affecting the analysis results. Duodenum and ileum, as parts of small intestine, also contain many intestinal microorganisms, which may affect poultry health and feed efficiency. Therefore, the main purpose of this study was to analyze the microbial composition and identify the relationship between FE and microbiota diversity in the duodenum and ileum of yellow broilers. We found that Actinobacteria, Bacteroidetes, Cyanobacteria, Firmicutes, and Proteobacteria were the dominant phyla in our experiment, consistent with the results reported by Xiao et al. (2017). The phylum Firmicutes had the highest relative abundance in the duodenum and ileum. In the duodenum, although the relative abundance of Firmicutes in the HFE group was higher than that in the LFE group, the opposite was observed in the ileum. Xiao et al. (2017) also reported that Firmicutes was the dominant phylum in the duodenum and ileum in domestic chickens, further validating our results. Moreover, we found that the genus *Lactobacillus* of the phylum Firmicutes was predominant in the duodenum and ileum. Furthermore, the relative abundances of Cyanobacteria and Actinobacteria were higher in the duodenum than in the ileum, whereas the relative abundance of Proteobacteria was higher in the ileum than in the duodenum. Thus, our findings demonstrated that there were differences in the microbiota of different intestinal segments at the phylum level, with varying trends in the dominant phyla in the HFE and LFE groups. Alpha diversity indices are used to estimate the diversity of microbial communities (Whittaker, 1972). Chao1 and Ace indices reflect the richness of microbial communities in samples, whereas Shannon and Simpson indices indicate the diversity of microbial communities; the latter being affected by richness and evenness. In our study, the gut microbiota was significantly more diverse in the duodenum than in the ileum, consistent with a previous study (Mu et al., 2017). The ileum is the main component of nutrient absorption, but it shows a low microbiota diversity (Bjerrum et al., 2006). In this study, the relative abundances of *Faecalibacterium* and *Ruminococcus* were higher in the duodenum than in the ileum, whereas the relative abundances of *SMB53* and *Enterococcus* were lower than those in the ileum. *SMB53* has been reported in human (Faria and Santos, 2020), bar-headed goose (Wang et al., 2016), pig (Yang et al., 2016) and mouse (Horie et al., 2017). *SMB53* is a poorly researched genus and belongs to anaerobic Clostridiaceae family, most members of which play a role in the consumption of intestinal mucus- and plant-derived saccharides such as glucose (Wüst et al., 2011). Biliary and pancreatic secretions promote the decomposition of nutrients in the short duodenum. While ileal epithelial cells are the main absorption sites of B vitamins and other nutrients, and the remaining nutrients cannot be further absorbed (Martinez-Guryn et al., 2019). Yang et al. (2016) have also reported that *SMB53* was significantly enriched in the ileum in pigs. Facultative anaerobic *Enterococcus* is ubiquitous in gastrointestinal tract of human and animals, and nature. The abundance and composition of microorganisms in the upper gastrointestinal tract are affected by swallowing air, transporting oxygen from host tissues and oxygenation through pancreatic and biliary secretions (Friedman et al., 2018). Different nutrient requirements may also affect the composition and abundance of microbiota in different intestinal segment. A previous study showed that increased *Lactobacillus* abundance reduces the diversity of microbial communities in the corresponding intestinal segments (Yan et al., 2017), consistent with the results of our study.

The small intestine is composed of the duodenum, jejunum, and ileum and is the site where most nutrients are digested and absorbed (Zhu et al., 2020). Through correlation analysis of duodenal and ileal microorganisms with FCR, we found that there were more negatively correlated bacteria than positively correlated bacteria at the phylum and genus levels. At the phylum level, the correlation between phylum and FCR was typically stronger in the ileum with coefficients −0.323 ∼ 0.195. The relative abundance of Firmicutes was the highest both in the duodenum and ileum in our study, which was also reported in chickens (Yan et al., 2017), human (Scott et al., 2013) and horses (Costa et al., 2015). Pearson correlation analysis showed that the phylum Firmicutes was positively correlated with FCR. The correlation of Firmicutes in the ileum was higher than that in the duodenum, which may be related to the higher proportion of Firmicutes in the ileum than in the duodenum. Cyanobacteria showed a very weak positive correlation in the duodenum and a very weak negative correlation in the ileum. Proteobacteria in the two intestinal segments also showed a very weak negative correlation. At the genus level, we found that *Lactobacillus* and *Faecalibacterium* had very weak positive correlations in the two intestinal segments *Lactobacillus* had a higher correlation in the ileum, whereas *Faecalibacterium* had a higher correlation in the duodenum, indicating that the higher relative abundances of these two bacteria were highly correlated with FCR. Notably, *Ruminococcus* also showed a very weak negative correlation with FCR. *SMB53* and *Enterococcus*, with increasing relative abundance in the ileum, showed slightly positive correlations with FCR in the duodenum. Correlations between FCR and genera *Paenibacillus*, *Thermus* and *Alcaligenes* were quite different in the duodenum and ileum. The relative abundance of *Thermus* were significantly higher in the HFE group than that of the LFE group in the ileum. There was no correlation between *Thermus* and FCR in the duodenum, while a slight negative correlation was found in the ileum. *Thermus*, a poorly studied genus in chickens, have been reported to be associated with feeding behavior in fish (Liu et al., 2019; Ying et al., 2020) and spiders (Hu et al., 2019). Digestive enzymes levels produced by *Thermus* changed as dietary sodium butyrate. In the process of polysaccharide digestion, gastrointestinal tract microbial community produces various short chain fatty acids (SCFA), such as acetate and butyrate (Yeoman et al., 2012). SCFA is an important nutrient for the host, which stimulates the proliferation of intestinal epithelial cells and the size of intestinal villi, thereby increasing the absorption surface area (Dibner and Richards, 2005). The small intestine is a site for digestion and absorption of nutrients, and bacteria in the small intestine use the same easily fermented nutrients as the host. Therefore, there is a competitive dietary nutrition relationship between the microbiota in the small intestine and the host (Apajalahti, 2005). Further research will be required to determine the exact contributions of microbiota to FE.

*Lactobacillus* was found to be the dominant bacterial genus in both the duodenum and ileum. In earlier studies, *Lactobacillus* was reported to be related to FCR in chickens (Torok et al., 2011; Stanley et al., 2016). Our experimental results also showed that the relative abundance of *Lactobacillus* was higher in the LFE group than in the HFE group, although the difference was not significant, which was also approved by Du et al. (2020). Research on the structure and variation of the intestinal microbiota in poultry can provide a theoretical basis for improving FE, growth performance, and the development of microbial biomass resources and preparations, which are essential for enhancing industrial efficiency. In previous works, a diet with *Lactobacillus* strains was found to increase BWG and FE and decrease mortality in broilers (Zulkifli et al., 2000; Timmerman et al., 2006). Additionally, Fonseca et al. (2010) suggested that supplementation with probiotics reduces the quantity of enterobacteria and pathogenic bacteria in the cecum of broiler chickens. However, *L. reuteri* L6798 and *L. reuteri* ATCC PTA 4659 were found to play opposite roles in weight change in mice (Fåk and Bäckhed, 2012). Therefore, further studies are needed to determine whether other *Lactobacillus* strains and genera can improve FE. In addition to the genera we identified, *Acinetobacter* and *Arcobacter* have also been reported to be predominant in broilers with high FCR, whereas microbes such as *Barnesiella* and *Cloacibacillus* were found to be abundant in broilers with low FCR (Singh et al., 2012). Wen et al. (2021) have demonstrated that lower abundances of duodenal *Akkermansia muciniphila* and cecal *Parabacteroides*, and higher abundances of cecal *Lactobacillus*, *Corynebacterium*, *Coprobacillus*, and *Slackia* were interrelated to better feed efficiency. Interestingly, *Akkermansia muciniphila*, as a member of phylum Verrucomicrobia, was also detected in the duodenum of our experimental broilers with extreme low relative abundance. *Akkermansia muciniphila* was isolated in feces of human (Derrien et al., 2004) and proved to be beneficial for improving obesity (Lukovac et al., 2014) and glucose tolerance (Greer et al., 2016). Since *Akkermansia muciniphila* had been proposed as a new functional microbe with probiotic properties, it can be used as a candidate bacterium to improve feed efficiency of birds.

With different genes and metabolic characteristics of gut microbes from those of the host, gut microbes are regarded as virtual organs involved in regulating the dynamic balance of host energy and regulating glucose and lipid metabolism (Salazar et al., 2014). Cani et al. (2007, 2008) reported that endotoxemia caused by bacterial lipopolysaccharide induced inflammatory response was associated with insulin resistance in diet-induced obesity mice. Intestinal microbiota also regulates glucose metabolism and lipid metabolism, affecting human health and weight (Karlsson et al., 2013). The increased *Lactobacillus* may be a consequence of increased intestinal glucose level. In this study, the abundance of *Lactobacillus* of the LFE group was higher than that of the HFE group both in the duodenum and ileum. Microbiota function prediction showed that the proportion of microorganisms involved in glucose metabolic process in the duodenum of the HFE and LFE groups was the highest among all differential pathways and was significantly enriched in the LFE group. Pathways including glycolysis II (from fructose 6-phosphate) and glycolysis I (from glucose 6-phosphate) were significantly enriched in the ileum of the LFE group. Microorganisms absorb nutrients through glycolysis, while competing with the host for nutrients, which will result in reduced FE in the host. Intestinal microbiota may balance the blood glucose in the host through gluconeogenesis. In summary, intestinal microorganisms affect the efficiency of host feed through multiple pathways.

## Conclusion

In conclusion, the results of this study show that the microbial community structures in the duodenum and ileum of yellow broilers in the HFE and LFE groups was similar. The relative abundance of different genera varied in duodenum and ileum and microbial diversity of duodenum was significantly higher than that of ileum. Moreover, the microbiota in the duodenum and ileum has different degrees of positive or negative correlations, and the ileal microbiota are more correlated with FE than the duodenal microbiota. Through differential functional analysis, we found that the gluconeogenesis pathway in the duodenum and the glycolysis pathway in the ileum may be related to reduced FE. The correlations between FE and microbiota in duodenum and ileum were at a low level. However, some genera, such as *Thermus*, *Faecalibacterium* and *Ruminococcus*, with differences in correlation in different intestinal segments and those with significant differences in the HFE and LFE groups in ileum deserve attention. However, in this study, we only focused on the microbial composition in the duodenum and ileum of yellow broilers with different FEs, and further isolation and culture experiments are needed to definitively demonstrate which bacteria can improve FE. These findings provide an important reference for formulating effective strategies to improve the growth performance and microbiota in the duodenum and ileum of yellow broilers.

## Data Availability Statement

The datasets presented in this study can be found in online repositories. The names of the repository/repositories and accession number(s) can be found below: https://www.ncbi.nlm.nih.gov/bioproject/PRJNA721286.

## Ethics Statement

The animal study was reviewed and approved by Animal Care Committee of China Agricultural University.

## Author Contributions

SC designed the experiments and edited the manuscript. HL conducted the data analysis and wrote the manuscript. YH collected and analyzed the data. TW and SZ processed and visualized the images. ZH edited the manuscript. All authors approved the final manuscript.

## Conflict of Interest

The authors declare that the research was conducted in the absence of any commercial or financial relationships that could be construed as a potential conflict of interest.

## Publisher’s Note

All claims expressed in this article are solely those of the authors and do not necessarily represent those of their affiliated organizations, or those of the publisher, the editors and the reviewers. Any product that may be evaluated in this article, or claim that may be made by its manufacturer, is not guaranteed or endorsed by the publisher.

## Supplementary Material

Supplementary Figure 1 Distribution of feed conversion ratio (FCR) value for each sample **(A)** and boxplot of FCR values in HFE and LFE groups **(B)**. HFE and LFE, high and low feed efficiency; NS, unselected samples; *^∗∗∗^P* < 0.001.

Supplementary Figure 2 Venn diagram of operational taxonomic units distribution in each group **(A)** and rarefaction curve for each sample **(B)**. *Hdu* and *Ldu*: high and low feed efficiency groups in the duodenum; Hil and Lil, high and low feed efficiency groups in the ileum.

Supplementary Table 1 Ingredients and nutrient composition of the feed used.

Supplementary Table 2 Difference analysis of relative abundance at phylum and genus levels of part of the microbes in the duodenum and ileum of HFE and LFE groups.

Supplementary Table 3 Statistics of Alpha diversity index of duodenal and ileal microbes.

Supplementary Table 4 Comparison of Meta-Cyc pathway differences of the duodenal and ileal microbes in the HFE and LFE groups.
