# Supplementary figures and images for: Microbial Composition in the Duodenum and Ileum of Yellow Broilers With High and Low Feed Efficiency
Source: Front Microbiol. 2021 Jul 27;12:689653. doi: 10.3389/fmicb.2021.689653 (PMC8353196; doi:10.3389/fmicb.2021.689653)

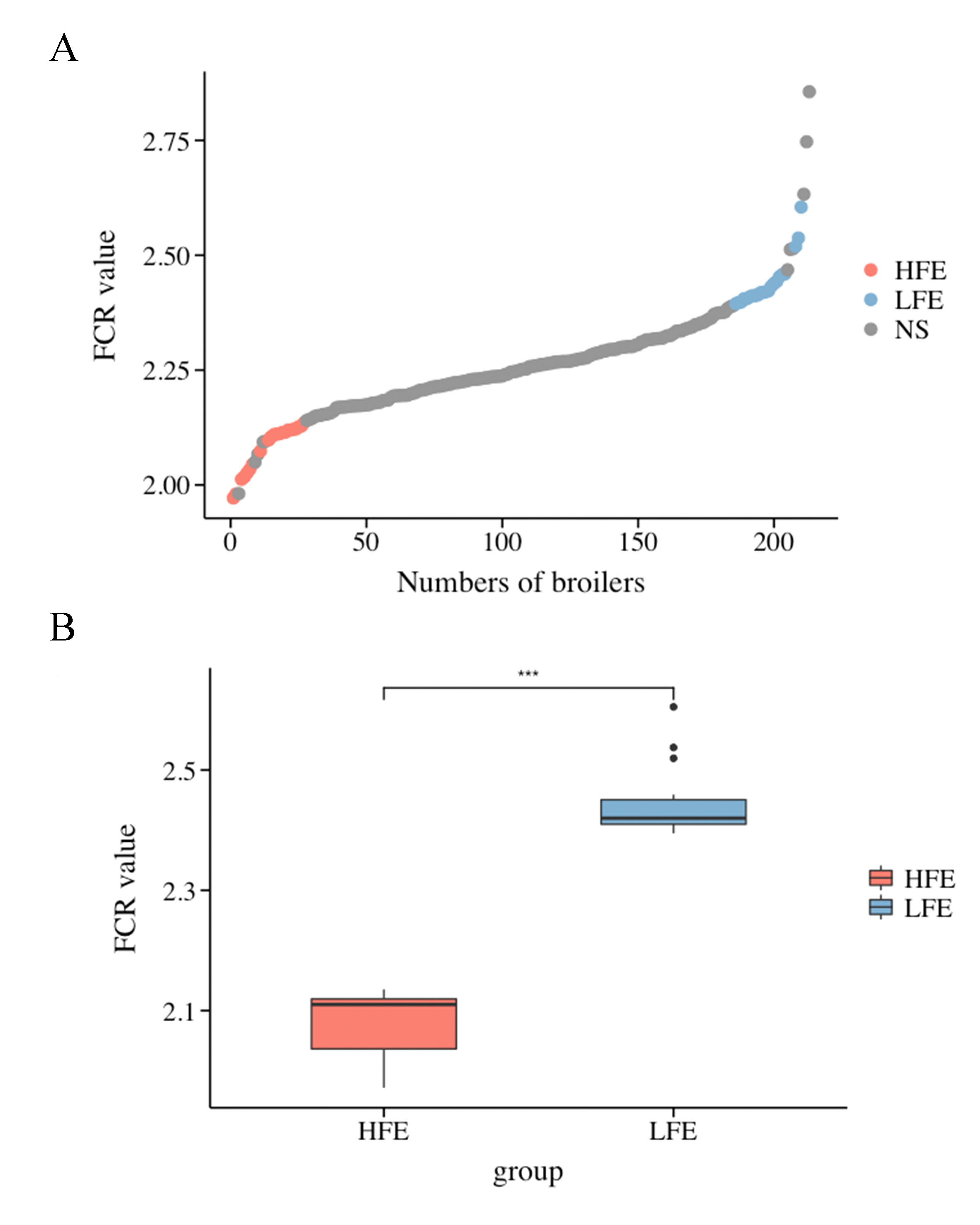

Supplement: Supplementary Figure 1 — Distribution of feed conversion ratio (FCR) value for each sample (A) and boxplot of FCR values in HFE and LFE groups (B). HFE and LFE, high and low feed efficiency; NS, unselected samples; ∗∗∗P < 0.001. [file Image_1.JPEG]

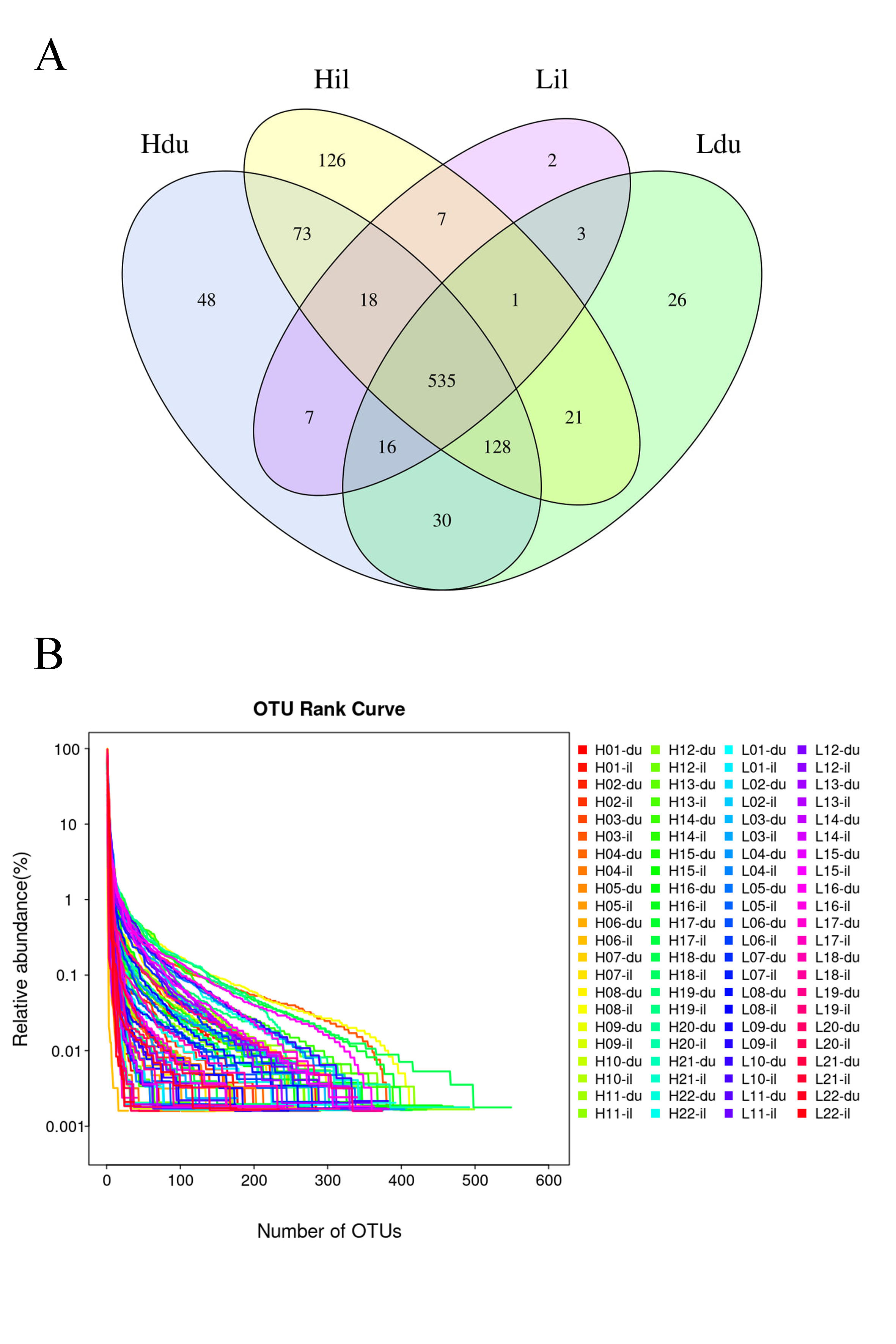

Supplement: Supplementary Figure 2 — Venn diagram of operational taxonomic units distribution in each group (A) and rarefaction curve for each sample (B). Hdu and Ldu: high and low feed efficiency groups in the duodenum; Hil and Lil, high and low feed efficiency groups in the ileum. [file Image_2.JPEG]
